# Poly[di­ethyl­ammonium [tetra-μ_2_-cyanido-κ^8^ *C*:*N*-tricuprate(I)]], a two-dimensional network solid

**DOI:** 10.1107/S2414314620009682

**Published:** 2020-07-21

**Authors:** Peter W. R. Corfield, Thomas James Stavola

**Affiliations:** a Department of Chemistry, Fordham University, 441 East Fordham Road, Bronx, NY 10458, USA; University of Kentucky, USA

**Keywords:** crystal structure, network, two-dimensional framework, copper cyanide, di­ethyl­ammonium, hydrogen bond

## Abstract

The title compound, (C_4_H_12_N)[Cu_3_(CN)_4_]_*n*_, crystallizes as a CuCN network solid, with di­ethyl­ammonium cations sandwiched between planar CuCN sheets comprised of trigonal planar and digonally coordinated Cu^I^ atoms bridged by linear CN groups to form 24-membered rings. The digonally coordinated Cu^I^ atoms and the di­ethyl­ammonium cations lie on separate crystallographic twofold rotation axes. One of the two independent CN groups has a 50:50 disordered orientation, while the other has one orientation favored due to a N—H⋯NC hydrogen bond between the di­ethyl­ammonium cation and the anionic CuCN framework. These hydrogen bonds link the sheets together into a three-dimensional network.

## Abstract 2

The title compound crystallizes as a CuCN network solid, with di­ethyl­ammonium cations sandwiched between two-dimensional, planar CuCN sheets comprised of trigonally and digonally coordinated Cu atoms in 24-membered rings.

## Graphical abstract

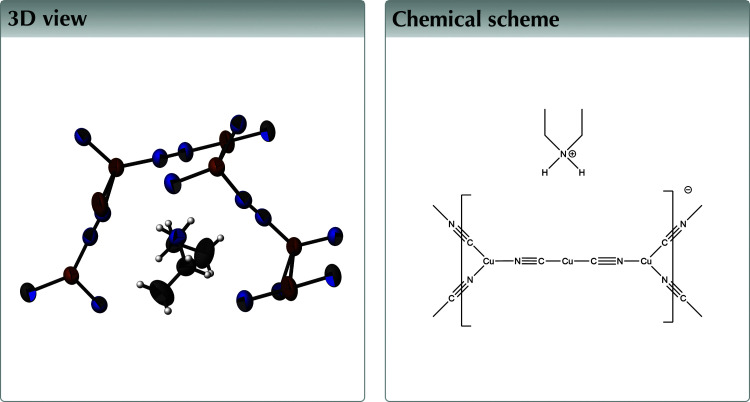

## Structure description

There has been continuing inter­est in the synthesis and structures of CuCN network solids containing protonated nitro­gen bases, with at least 40 such structures listed in the CSD (Groom *et al.*, 2016 ▸). For instance, a recent paper reports optical memory effects for two tetra­methyl­ammonium CuCN structures (Nicholas *et al.*, 2019 ▸) while Grifasi *et al.* (2016 ▸) is one of several papers reporting on the inter­esting topologies and photoluminescence of many CuCN networks. The present compound was prepared as part of our own ongoing structural studies in this area.

**Figure 1:**
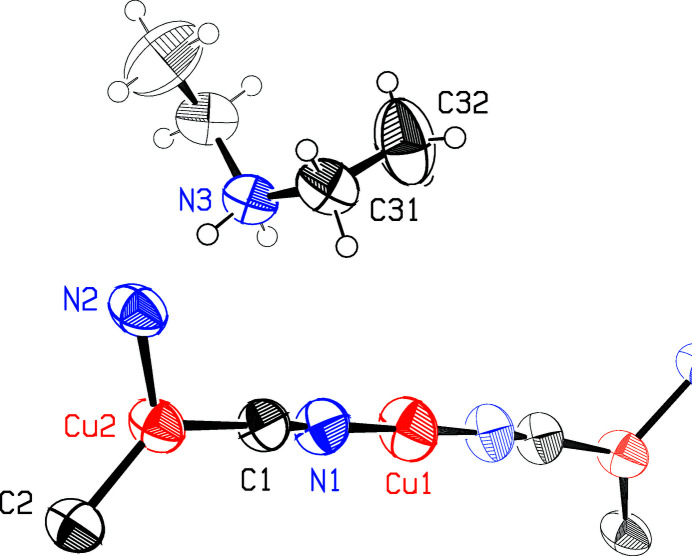
The asymmetric unit of the title compound is emboldened. Different crystallographic twofold axes pass through Cu1 and N3. Displacement ellipsoids are drawn at the 50% probability level, while H atoms are depicted as arbitrary spheres. Cu atoms are colored red, N atoms blue, and C and H atoms black.

**Figure 2:**
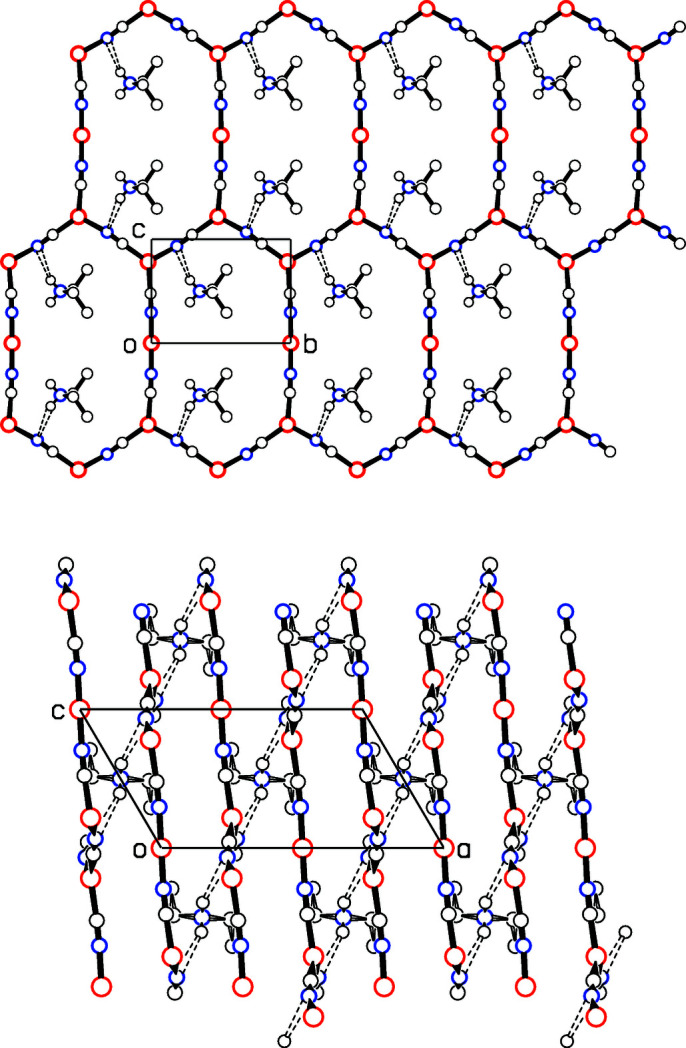
Top: View along the *a* axis of one sheet. Hydrogen bonds from cations above and below the sheet are shown. Bottom: Projection down the *b* axis, showing the sheets stacked perpendicular to the *a* axis, and the hydrogen bonds linking the sheets together. (Scale is slightly larger than in the top diagram.) The chains of hydrogen bonds along the [102] direction are evident. Cu atoms are colored red, N atoms blue, and C and NH atoms black. H atoms of the C_2H_5__ gropu are not shown, and only one orientation for each of the disordered CN groups is drawn.

Of the two independent Cu atoms, Cu1 is linearly coordinated to two CN groups and lies on the crystallographic twofold rotation axis [0, *y*, 0], while trigonally coordinated Cu2 is in a general position, Fig. 1 ▸. Each of the two independent CN groups bridges two copper(I) atoms to build a two-dimensional CuCN network perpendicular to the *a* axis. Four such sheets cross the unit cell, as shown in the packing diagram, Fig. 2 ▸. The network is made up of 24-membered rings, which are almost planar, with an r.m.s. deviation from the 24-atom plane of 0.128 (5) Å, where the e.s.d. given is the average of the 24 individual e.s.d.’s. Most such networks in the literature are honeycomb structures made up of 18-membered hexa­gonal rings, although a network similar to that described here was reported by Ferlay *et al.* (2013 ▸). The three-coordinated Cu2 atom has a geometry far from ideal trigonal planar, with C/N—Cu—C/N angles of 114.7 (3), 116.4 (2), and 128.3 (3)° and bond lengths Cu—C/N ranging from 1.889 (8) to 1.960 (7) Å.

The ammonium cation lies on the crystallographic twofold axis [0, *y*, 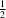 ] and assumes a *gauche* conformation, with the torsion angle C32—C31—N3—C31(−*x*, *y*, 1 − *z*) = −62.1 (6)°. Each cation forms two N—H⋯N hydrogen bonds to N2 of the bridging C2≡N2 group of two adjacent sheets, which ties adjacent sheets into a three-dimensional network, as shown in Fig. 2 ▸. Table 1 ▸ gives details of the single independent hydrogen bond, while the lower part of Fig. 2 ▸ reveals that the hydrogen bonds in the crystal point along the [102] direction.

## Synthesis and crystallization

A mixture of 0.359 g (4.01 mmol) of CuCN and 0.330 g of NaCN (6.73 mmol) with 25 ml of H_2_O was stirred and the light remaining precipitate was filtered off. 1.55 g (21.2 mmol) of di­ethyl­amine dissolved in 10 ml of H_2_O were added, and the stirred mixture was left open to air. Crystals began to form after one week and were harvested as conglomerates of thick, yellow–green plates several weeks later. The intent had been to prepare a mixed-valence compound similar to those prepared from bidentate amines (Corfield & Michalski, 2014 ▸; Corfield & Sabatino, 2017 ▸) and to use the fivefold excess of base to stabilize any Cu^II^ formed by air-oxidation. However, no crystalline mixed-valence compounds containing the base were obtained in this and similar preparations with di­ethyl­amine. The IR spectrum, obtained with a Thermo Scientific Nicolet iS50 FT–IR instrument, showed strong stretching bands at 2111 cm^−1^ and 2136 cm^−1^ for CN, and at 3118 cm^−1^ and 3186 cm^−1^ for N—H. The N—H frequencies for the protonated base may be compared with the band at 3281 cm^−1^ (*w*) found for the free base di­ethyl­amine.

## Refinement

Crystal data, data collection and structure refinement details are summarized in Table 2 ▸. Towards the end of the refinements, each of the two CN groups was refined as a superposition of NC and CN groups, whose occupancies were varied. For C1≡N1, the occupancy factor refined to close to 50%, so this occupancy was fixed at 50%, while the occupancy for C2≡N2 favors one orientation over the other by 78 (8)%. This preferred orientation is doubtless due to the hydrogen-bonding inter­actions with the cation discussed above. The Flack *x* factor (Parsons *et al.*, 2013 ▸) is 0.096 (25), which implies that the crystal exhibits minor twinning about the (010) plane; more pronounced twinning was seen in a different crystal not used in this work. The final refinement uses the *SHELXL* BASF and TWIN commands, with no noticeable changes in the structure.

## Supplementary Material

Crystal structure: contains datablock(s) I. DOI: 10.1107/S2414314620009682/pk4028sup1.cif

Structure factors: contains datablock(s) I. DOI: 10.1107/S2414314620009682/pk4028Isup2.hkl

CCDC reference: 2016688

Additional supporting information:  crystallographic information; 3D view; checkCIF report

## Figures and Tables

**Table 1:** Hydrogen-bond geometry (Å, °)

*D*—H⋯*A*	*D*—H	H⋯*A*	*D*⋯*A*	*D*—H⋯*A*
N3—H3⋯N2	0.89 (1)	2.44 (4)	3.230 (6)	149 (6)

**Table 2:** Experimental details

Crystal data	
Chemical formula	(C_4_H_12_N)[Cu_3_(CN)_4_]
*M*_r_	368.85
Crystal system, space group	Monoclinic, *C*2
Temperature (K)	302
*a*, *b*, *c* (Å)	12.6825 (8), 8.3355 (5), 7.2205 (5)
β (°)	120.444 (3)
*V* (Å^3^)	658.07 (8)
*Z*	2
Radiation type	Mo *K*α
μ (mm^−1^)	4.78
Crystal size (mm)	0.15 × 0.13 × 0.08

Data collection	
Diffractometer	Enraf–Nonius KappaCCD
Absorption correction	Multi-scan (Otwinowski & Minor,1997 ▸)
*T*_min_, *T*_max_	0.51, 0.68
No. of measured, independent and observed [*I* > 2σ(*I*)] reflections	2500, 1487, 1191
*R*_int_	0.039
(sin θ/λ)_max_ (Å^−1^)	0.648

Refinement	
*R*[*F* ^2^ > 2σ(*F* ^2^)], *wR*(*F* ^2^), *S*	0.031, 0.069, 1.04
No. of reflections	1487
No. of parameters	82
No. of restraints	2
H-atom treatment	H atoms treated by a mixture of independent and constrained refinement
Δρ_max_, Δρ_min_ (e Å^−3^)	0.22, −0.29
Absolute structure	Twinning involves reflection, so the Flack parameter of 0.13 (5) implies the presence of a small amount of the inverted form
Absolute structure parameter	0.13 (5)

Computer programs: *KappaCCD Server Software* (Nonius, 1997 ▸), *DENZO* and *SCALEPACK* (Otwinowski & Minor, 1997 ▸), *SHELXS97* (Sheldrick, 2008 ▸), *SHELXL2017/1* (Sheldrick, 2015 ▸), *ORTEPIII* (Burnett & Johnson, 1996 ▸), *ORTEP-3 for Windows* (Farrugia, 2012 ▸) and *publCIF* (Westrip, 2010 ▸).

## full crystallographic data

### Crystal data

**Table:**

(C_4_H_12_N)[Cu_3_(CN)_4_]	*F*(000) = 364
*M**_r_* = 368.85	*D*_x_ = 1.861 Mg m^−^^3^*D*_m_ = 1.84 (1) Mg m^−^^3^*D*_m_ measured by flotation in CCl4/dibromoethane mixtures
Monoclinic, *C*2	Mo *K*α radiation, λ = 0.7107 Å
Hall symbol: C 2y	Cell parameters from 791 reflections
*a* = 12.6825 (8) Å	θ = 3.3–27.4°
*b* = 8.3355 (5) Å	µ = 4.78 mm^−^^1^
*c* = 7.2205 (5) Å	*T* = 302 K
β = 120.444 (3)°	Irregular block, pale green
*V* = 658.07 (8) Å^3^	0.15 × 0.13 × 0.08 mm
*Z* = 2	

### Data collection

**Table:**

Enraf–Nonius KappaCCD diffractometer	1487 independent reflections
Radiation source: fine-focus sealed tube	1191 reflections with *I* > 2σ(*I*)
Graphite monochromator	*R*_int_ = 0.039
Detector resolution: 9 pixels mm^-1^	θ_max_ = 27.4°, θ_min_ = 3.3°
combination of ω and φ scans	*h* = −16→16
Absorption correction: multi-scan (Otwinowski & Minor,1997)	*k* = −10→10
*T*_min_ = 0.51, *T*_max_ = 0.68	*l* = −9→9
2500 measured reflections	

### Refinement

**Table:**

Refinement on *F*^2^	Hydrogen site location: mixed
Least-squares matrix: full	H atoms treated by a mixture of independent and constrained refinement
*R*[*F*^2^ > 2σ(*F*^2^)] = 0.031	*w* = 1/[σ^2^(*F*_o_^2^) + (0.021*P*)^2^ + 0.730*P*] where *P* = (*F*_o_^2^ + 2*F*_c_^2^)/3
*wR*(*F*^2^) = 0.069	(Δ/σ)_max_ < 0.001
*S* = 1.04	Δρ_max_ = 0.22 e Å^−^^3^
1487 reflections	Δρ_min_ = −0.29 e Å^−^^3^
82 parameters	Extinction correction: SHELXL-2017/1 (Sheldrick 2015), Fc^*^=kFc[1+0.001xFc^2^λ^3^/sin(2θ)]^-1/4^
2 restraints	Extinction coefficient: 0.015 (3)
Primary atom site location: heavy-atom method	Absolute structure: Twinning involves reflection, so the Flack parameter of 0.13 (5) implies the presence of a small amount of the inverted form
Secondary atom site location: difference Fourier map	Absolute structure parameter: 0.13 (5)

### Special details

**Table:**

Geometry. All esds (except the esd in the dihedral angle between two l.s. planes) are estimated using the full covariance matrix. The cell esds are taken into account individually in the estimation of esds in distances, angles and torsion angles; correlations between esds in cell parameters are only used when they are defined by crystal symmetry. An approximate (isotropic) treatment of cell esds is used for estimating esds involving l.s. planes.
Refinement. Hydrogen atoms on the C atoms were constrained, with C—H distances of 0.97 Å for the methylene group and 0.96 Å for the methyl group. The N—H atom was refined, with a restraint on the N—H bond length but not on the temperature factor.

### Fractional atomic coordinates and isotropic or equivalent isotropic displacement parameters (Å^2^)

**Table:**

	*x*	*y*	*z*	*U*_iso_*/*U*_eq_	Occ. (<1)
Cu1	0.000000	0.0002 (2)	0.000000	0.0860 (6)	
Cu2	0.18717 (6)	−0.02348 (14)	0.78258 (9)	0.0636 (3)	
C1	0.1211 (4)	−0.0060 (10)	0.4775 (7)	0.0605 (12)	0.5
N1A	0.1211 (4)	−0.0060 (10)	0.4775 (7)	0.0605 (12)	0.5
N1	0.0777 (4)	−0.0015 (11)	0.2956 (8)	0.0683 (16)	0.5
C1A	0.0777 (4)	−0.0015 (11)	0.2956 (8)	0.0683 (16)	0.5
C2A	0.2142 (6)	0.1819 (7)	0.9323 (10)	0.066 (2)	0.22 (8)
N2	0.2142 (6)	0.1819 (7)	0.9323 (10)	0.066 (2)	0.78 (8)
N2A	0.2525 (7)	0.2919 (8)	1.0428 (11)	0.064 (2)	0.22 (8)
C2	0.2525 (7)	0.2919 (8)	1.0428 (11)	0.064 (2)	0.78 (8)
N3	0.000000	0.3473 (8)	0.500000	0.0606 (17)	
H3	0.033 (6)	0.277 (6)	0.606 (8)	0.09 (2)*	
C31	0.0968 (5)	0.4390 (8)	0.4846 (11)	0.071 (2)	
H31A	0.140222	0.506523	0.610650	0.106*	
H31B	0.155145	0.363683	0.484731	0.106*	
C32	0.0488 (8)	0.5391 (14)	0.2931 (14)	0.118 (4)	
H32A	0.115278	0.590203	0.288774	0.177*	
H32B	−0.004388	0.619311	0.297044	0.177*	
H32C	0.003720	0.473602	0.167536	0.177*	

### Atomic displacement parameters (Å^2^)

**Table:**

	*U*^11^	*U*^22^	*U*^33^	*U*^12^	*U*^13^	*U*^23^
Cu1	0.0970 (8)	0.0960 (16)	0.0395 (5)	0.000	0.0158 (5)	0.000
Cu2	0.0685 (4)	0.0588 (4)	0.0457 (3)	0.0009 (4)	0.0157 (3)	0.0021 (4)
C1	0.057 (2)	0.068 (3)	0.047 (2)	0.007 (3)	0.0188 (19)	0.003 (3)
N1A	0.057 (2)	0.068 (3)	0.047 (2)	0.007 (3)	0.0188 (19)	0.003 (3)
N1	0.065 (3)	0.081 (5)	0.045 (2)	0.008 (3)	0.017 (2)	−0.001 (3)
C1A	0.065 (3)	0.081 (5)	0.045 (2)	0.008 (3)	0.017 (2)	−0.001 (3)
C2A	0.079 (4)	0.051 (4)	0.050 (3)	0.000 (3)	0.020 (3)	0.002 (3)
N2	0.079 (4)	0.051 (4)	0.050 (3)	0.000 (3)	0.020 (3)	0.002 (3)
N2A	0.074 (4)	0.053 (4)	0.047 (4)	0.004 (3)	0.016 (3)	0.007 (3)
C2	0.074 (4)	0.053 (4)	0.047 (4)	0.004 (3)	0.016 (3)	0.007 (3)
N3	0.056 (4)	0.056 (4)	0.062 (4)	0.000	0.023 (3)	0.000
C31	0.054 (3)	0.067 (6)	0.077 (4)	0.001 (3)	0.023 (3)	0.006 (3)
C32	0.089 (5)	0.158 (12)	0.097 (6)	0.007 (6)	0.040 (5)	0.049 (6)

### Geometric parameters (Å, º)

**Table:**

Cu1—N1^i^	1.842 (5)	N3—C31^iii^	1.497 (7)
Cu1—N1	1.842 (5)	N3—H3	0.885 (14)
Cu2—N2A^ii^	1.889 (7)	C31—C32	1.457 (10)
Cu2—C1	1.925 (4)	C31—H31A	0.9700
Cu2—N2	1.960 (7)	C31—H31B	0.9700
C1—N1	1.139 (6)	C32—H32A	0.9600
N2—C2	1.149 (7)	C32—H32B	0.9600
N3—C31	1.497 (7)	C32—H32C	0.9600
			
N1^i^—Cu1—N1	179.1 (6)	C32—C31—H31A	108.9
N2A^ii^—Cu2—C1	128.3 (3)	N3—C31—H31A	108.9
N2A^ii^—Cu2—N2	116.4 (2)	C32—C31—H31B	108.9
C1—Cu2—N2	114.7 (3)	N3—C31—H31B	108.9
N1—C1—Cu2	176.5 (6)	H31A—C31—H31B	107.7
C1—N1—Cu1	176.8 (6)	C31—C32—H32A	109.5
C2—N2—Cu2	167.0 (6)	C31—C32—H32B	109.5
N2—C2—Cu2^iv^	178.3 (5)	H32A—C32—H32B	109.5
C31—N3—C31^iii^	118.6 (7)	C31—C32—H32C	109.5
C31—N3—H3	111 (5)	H32A—C32—H32C	109.5
C31^iii^—N3—H3	109 (5)	H32B—C32—H32C	109.5
C32—C31—N3	113.5 (5)		

Symmetry codes: (i) −*x*, *y*, −*z*; (ii) −*x*+1/2, *y*−1/2, −*z*+2; (iii) −*x*, *y*, −*z*+1; (iv) −*x*+1/2, *y*+1/2, −*z*+2.

### Hydrogen-bond geometry (Å, º)

**Table:**

*D*—H···*A*	*D*—H	H···*A*	*D*···*A*	*D*—H···*A*
N3—H3···N2	0.89 (1)	2.44 (4)	3.230 (6)	149 (6)

## References

[bb1] Burnett, M. N. & Johnson, C. K. (1996). *ORTEPIII*. Report ORNL6895. Oak Ridge National Laboratory, Tennessee, USA.

[bb2] Corfield, P. W. R. & Michalski, J. F. (2014). *Acta Cryst.* E**70**, m76–m77. 10.1107/S160053681400172X PMC3998273 24764834

[bb3] Corfield, P. W. R. & Sabatino, A. (2017). *Acta Cryst.* E**73**, 141–146. 10.1107/S2056989017000111 PMC5290552 28217329

[bb4] Farrugia, L. J. (2012). *J. Appl. Cryst.* **45**, 849–854.

[bb5] Ferlay, S., Dechambenoit, P., Kyritsakas, N. & Hosseini, M. W. (2013). *Dalton Trans.* **42**, 11661–11671. 10.1039/c3dt51252e 23824217

[bb6] Grifasi, F., Priola, E., Chierotti, M. R., Diana, E., Garino, C. & Gobetto, R. (2016). *Eur. J. Inorg. Chem.* pp. 2975–2983.

[bb7] Groom, C. R., Bruno, I. J., Lightfoot, M. P. & Ward, S. C. (2016). *Acta Cryst.* B**72**, 171–179. 10.1107/S2052520616003954 PMC4822653 27048719

[bb8] Nicholas, A. D., Bullard, R. M., Wheaton, A. M., Streep, M., Nicholas, V. A., Pike, R. D. & Patterson, H. H. (2019). *Materials*, **12**, 1211–1229. 10.3390/ma12081211 PMC6514951 31013868

[bb9] Nonius (1997). *KappaCCD Server Software*. Nonius BV, Delft, The Netherlands.

[bb10] Otwinowski, Z. & Minor, W. (1997). *Methods in Enzymology*, Vol. 276, *Macromolecular Crystallography*, Part A, edited by C. W. Carter Jr & R. M. Sweet, pp. 307–326. New York: Academic Press. 10.1016/S0076-6879(97)76066-X 27754618

[bb11] Parsons, S., Flack, H. D. & Wagner, T. (2013). *Acta Cryst.* B**69**, 249–259. 10.1107/S2052519213010014 PMC3661305 23719469

[bb12] Sheldrick, G. M. (2008). *Acta Cryst*. A**64**, 112–122. 10.1107/S0108767307043930 18156677

[bb13] Sheldrick, G. M. (2015). *Acta Cryst.* C**71**, 3–8.

[bb14] Westrip, S. P. (2010). *J. Appl. Cryst.* **43**, 920–925.

